# Stratified Care vs Stepped Care for Depression: A Cluster Randomized Clinical Trial

**DOI:** 10.1001/jamapsychiatry.2021.3539

**Published:** 2021-12-08

**Authors:** Jaime Delgadillo, Shehzad Ali, Kieran Fleck, Charlotte Agnew, Amy Southgate, Laura Parkhouse, Zachary D. Cohen, Robert J. DeRubeis, Michael Barkham

**Affiliations:** 1 Clinical and Applied Psychology Unit, Department of Psychology, University of Sheffield, Sheffield, United Kingdom; 2 Rotherham Doncaster and South Humber NHS Foundation Trust, Doncaster, United Kingdom; 3 Department of Epidemiology and Biostatistics, Schulich School of Medicine and Dentistry, Western University, London, Ontario, Canada; 4 Institute of Mental Health Policy Research, Centre for Addictions and Mental Health, Toronto, Ontario, Canada; 5 Mental Health and Addictions Research Group, Department of Health Sciences, University of York, York, United Kingdom; 6 Department of Psychology, Macquarie University, Sydney, Australia; 7 Lancashire and South Cumbria NHS Foundation Trust, Preston, United Kingdom; 8 Department of Psychiatry, University of California, Los Angeles; 9 Department of Psychology, University of Pennsylvania, Philadelphia

## Abstract

**Importance:**

Depression is a major cause of disability worldwide. Although empirically supported treatments are available, there is scarce evidence on how to effectively personalize psychological treatment selection.

**Objective:**

To compare the clinical effectiveness and cost-effectiveness of 2 treatment selection strategies: stepped care and stratified care.

**Design, Setting, and Participants:**

This multisite, cluster randomized clinical trial recruited participants from the English National Health Service from July 5, 2018, to February 1, 2019. Thirty clinicians working across 4 psychological therapy services were randomly assigned to provide stratified (n = 15) or stepped (n = 15) care. In stepped care, patients sequentially access low-intensity guided self-help followed by high-intensity psychotherapy. In stratified care, patients are matched with either low- or high-intensity treatments at initial assessment. Data were analyzed from May 18, 2020, to October 13, 2021, using intention-to-treat principles.

**Interventions:**

All clinicians used the same interview schedule to conduct initial assessments with patients seeking psychological treatment for common mental disorders, but those in the stratified care group received a personalized treatment recommendation for each patient generated by a machine learning algorithm. Eligible patients received either stratified or stepped care (ie, treatment as usual).

**Main Outcomes and Measures:**

The preregistered outcome was posttreatment reliable and clinically significant improvement (RCSI) of depression symptoms (measured using the 9-item Patient Health Questionnaire). The RCSI outcome was compared between groups using logistic regression adjusted for baseline severity. Cost-effectiveness analyses compared incremental costs and health outcomes of the 2 treatment pathways.

**Results:**

A total of 951 patients were included (618 women among 950 with data available [65.1%]; mean [SD] age, 38.27 [14.53] years). The proportion of cases of RCSI was significantly higher in the stratified care arm compared with the stepped care arm (264 of 505 [52.3%] vs 134 of 297 [45.1%]; odds ratio, 1.40 [95% CI, 1.04-1.87]; *P* = .03). Stratified care was associated with a higher mean additional cost per patient (£104.5 [95% CI, £67.5-£141.6] [$139.83 (95% CI, $90.32-$189.48)]; *P* < .001) because more patients accessed high-intensity treatments (332 of 583 [56.9%] vs 107 of 368 [29.1%]; χ^2^ = 70.51; *P* < .001), but this additional cost resulted in an approximately 7% increase in the probability of RCSI.

**Conclusions and Relevance:**

In this cluster randomized clinical trial of adults with common mental disorders, stratified care was efficacious and cost-effective for the treatment of depression symptoms compared with stepped care. Stratified care can improve depression treatment outcomes at a modest additional cost.

**Trial Registration:**

isrctn.org Identifier: ISRCTN11106183

## Key Points

**Question:**

Is stratified care an efficacious and cost-effective approach to psychological treatment selection compared with stepped care?

**Findings:**

In this cluster randomized clinical trial of 951 adults with common mental disorders, stratified care was efficacious and cost-effective for the treatment of depression symptoms relative to stepped care.

**Meaning:**

These findings suggest that stratified care has the potential to improve depression treatment outcomes at a modest incremental cost.

## Teaser

This cluster randomized clinical trial assesses whether stratified care is effective and cost-effective compared with usual stepped care among patients seeking psychological treatment for common mental disorders, including depression.

## Introduction

Clinical guidelines for the management of depression recommend psychological interventions organized in a stepped care model, in which most patients access only low-intensity treatments such as guided self-help, and patients who remain symptomatic after this step can access more intensive and costly psychotherapies such as cognitive behavioral therapy.^1^ Systematic reviews of clinical trials indicate that stepped care results in improved effect sizes (Cohen *d* = 0.34)^2^ and higher odds of recovery (odds ratio [OR], 1.31 [IQR, 1.05-1.66])^3^ relative to usual care. In theory, stepped care is a self-correcting model^4^ in which patients eventually receive an appropriately intensive treatment for their needs. This model widens access to care by offering the least restrictive and least costly interventions to most people.^5^

Stepped care has been implemented at a national level in England, through the Improving Access to Psychological Therapies (IAPT) program, which currently receives more than 1 million referrals per year.^6^ A systematic review of studies arising from the IAPT program^7^ indicated that stepped care is generally associated with large pre- to posttreatment effect sizes for depression (Cohen *d* = 0.87). However, these effects were attenuated in subgroups of patients with more complex presentations,^7^ such as those with comorbid physical illnesses,^8^ personality disorder traits,^9^ disabilities,^10^ and low treatment expectancies^10^ and those living in socioeconomically deprived circumstances.^11^ These complicating factors have a cumulative effect, such that patients with several of these features tend to have poorer treatment outcomes.^10,12^ On this basis, some have argued that IAPT services should move toward a stratified approach to psychological treatment selection, which would involve matching the intensity of treatment to the level of complexity in each individual case.^12^

Stratified medicine aims to identify individuals who will have the most clinical benefit or least harm from specific treatments.^13^ Recent studies^10,12,14,15^ have indicated that stratified care has potential to improve the effectiveness of psychological care for depression. However, most of these studies draw their conclusions from post hoc analyses of retrospective data, and the only prospective study^16^ was underpowered to test its primary hypothesis. Rigorous and adequately powered experimental studies are necessary to determine whether stratified care may be an effective and affordable way to organize psychological interventions. To fill this evidence gap, we conducted a cluster randomized clinical trial of stratified care vs stepped care in IAPT services. We hypothesized that stratified care would improve depression treatment outcomes compared with stepped care and that this would be explained by higher improvement rates in complex cases matched with high-intensity treatment.

## Methods

### Study Design

This pragmatic, multisite, single-blind, cluster randomized clinical trial involved 4 IAPT services in northern England that were managed by Lancashire and South Cumbria NHS (National Health Service) Foundation Trust and Rotherham Doncaster and South Humber NHS Foundation Trust. These services implemented stepped care in line with national guidelines.^1^ The trial protocol (Supplement 1) was preregistered and was approved by a research ethics committee and the Health Research Authority. This study followed the Consolidated Standards of Reporting Trials (CONSORT) reporting guideline.

### Participants

The research team recruited clinicians after presenting the study rationale at clinical team meetings. Interested clinicians provided written informed consent via email. Patients seeking psychological treatment were recruited by participating clinicians using a standardized recruitment script at the start of routine assessments that aimed to determine suitability for treatment in the IAPT program. Patients provided verbal consent because assessments were conducted via telephone.

Clinicians were included if they were psychological well-being practitioners who conducted initial assessments in the participating services and were qualified with a nationally recognized postgraduate certificate in low-intensity psychological interventions (eMethods 1 in Supplement 2). Consenting patients were eligible if they (1) sought treatment for a common mental disorder (unipolar depression, posttraumatic stress disorder, obsessive-compulsive disorder, body dysmorphic disorder, phobias, and other anxiety disorders); (2) were deemed suitable for treatment in the IAPT program according to clinical guidelines; and (3) accessed treatment, defined by attending at least 1 session after their initial assessment. Regarding the second criterion, patients deemed unsuitable for treatment in this setting had severe mental disorders (eg, psychotic, bipolar), severe learning disabilities, substance dependence, acute suicidal risk, or problems not meeting criteria for a common mental disorder.^17^ Patients were excluded from these services and the study if they were already accessing psychological treatment elsewhere (ie, privately or through other services). No other exclusion criteria were applied, and patients were eligible for participation regardless of their current use of medications or other medical interventions.

### Randomization and Masking

Consenting clinicians were randomized to a stratified treatment group or a stepped care (treatment as usual) control group by an independent research assistant using a computer-generated 1:1 randomization schedule in blocks of 4, stratified by team. Randomization was clustered by clinicians to minimize contamination bias that may occur if clinicians applied stratified care with some patients and stepped care with others. Clinicians were therefore aware of their random allocation, which was communicated to them after randomization. Patients provided informed consent for clinicians to gather assessment information, enter it into a computer system, and use it to inform their treatment recommendation, but they were blinded to the decision-making process that guided each of the treatment groups.

### Procedures

#### Assessment Interviews

All consenting patients were assessed by participating clinicians using the same semistructured interview schedule. These were routine telephone-based assessments that lasted an average of 40 minutes and followed practice guidelines for IAPT services.^17^ The assessments covered the patient’s presenting problems and their impact, history, current life circumstances, and treatment goals. As part of this assessment, clinicians in both groups gathered clinical and demographic data that were entered in a computerized application as part of the study procedures. Race and ethnicity were self-reported by participants. Although they provided a self-reported category to clinicians who undertook the assessments, this information was aggregated in a binary variable (White British; other) by clinical services before data were shared with the research team. No other details about race and ethnicity were available to the research team.

Clinicians in the stratified care group used a version of the application that provided a personalized treatment recommendation in real time, recommending either a low- or high-intensity treatment based on each patient’s features. These clinicians were trained to discuss this recommendation with patients following good practice principles of shared decision-making^18^ and came to a final treatment allocation decision that was recorded in the application. Clinicians in the stepped care control group used the application only to enter data, but they did not receive a personalized recommendation, and they allocated patients to treatment following guidelines for stepped care.^1,17^ Consistent with these guidelines, stepped care initially allocates most patients to low-intensity treatments, but patients with specific disorders (eg, social anxiety disorder, posttraumatic stress disorder, body dysmorphic disorder) and those who have severe impairment can be referred directly for high-intensity treatments.^17^ Treatment allocation decisions in routine care are often made after initial assessment interviews with patients and in consultation with supervisors or senior clinicians.

#### Artificial Intelligence Technology

The stratified care application used in this trial is a technology that (1) collects data, (2) processes inputs using a machine learning algorithm, and (3) outputs a personalized treatment recommendation using automated decision rules. The inputs for the algorithm were patient-reported measures of depression,^19^ anxiety,^20^ functional impairment,^21^ personality traits,^22^ employment status, and race and ethnicity. The algorithm calculates an expected prognosis (ie, a probability of full remission of depression and anxiety symptoms after treatment) based on which cases are classified as standard (better expected prognosis) or complex (poorer expected prognosis). Standard cases are matched with low-intensity treatments, and patients later have the option to move to high-intensity treatment if necessary, whereas complex cases are matched directly with high-intensity treatments. The rationale is to offer more intensive treatments to patients with higher risk of poor treatment outcomes, consistent with principles of stratified medicine.^13^ Further technical details about the data sources, machine learning approach (LASSO [least absolute shrinkage and selection operator] with optimal scaling), model development, and external cross-validation are available elsewhere.^12^ In addition, the stratified care application was programmed to implement decision rules that would ensure compliance with national clinical guidelines^1^ for the treatment allocation of patients with the aforementioned disorders that are treated only with high-intensity psychotherapies in the IAPT program.^17^ As such, this treatment selection approach was designed to fast-track 2 groups of patients to high-intensity treatments: patients with specific conditions for which only psychotherapy is indicated and patients whose cases are classified as complex.

#### Psychological Interventions

After initial assessment interviews, patients accessed their assigned interventions with the first available clinician in each service (the treating clinician was not the same person as the assessing clinician). Low-intensity interventions are based on principles of cognitive behavioral therapy and involve learning coping skills with the support of a qualified psychological well-being practitioner^23^ for up to 8 sessions (each lasting 30 minutes). Low-intensity interventions can be delivered as individual-guided self-help, in group settings, or as telephone-guided computerized cognitive behavioral therapy. High-intensity interventions are lengthier (≤20 one-hour sessions) evidence-based psychotherapies including cognitive behavioral therapy, person-centered experiential counseling for depression, and eye movement desensitization and reprocessing for posttraumatic stress disorder. These interventions were delivered by clinicians qualified to a postgraduate level, following structured treatment protocols endorsed by national guidelines,^23,24^ and under regular supervision (equivalent of 1 h/wk). Consistent with the pragmatic trial design, we did not record, monitor, or modify these interventions in any way to preserve the integrity of routinely delivered psychological care.

#### Training

All participating clinicians attended a 2-hour training course that covered the study design, informed consent and recruitment tasks, and data collection tasks. Clinicians randomized to the stratified care group attended 1 additional hour of training (3 hours in total), which covered the stratified care algorithm, its decision-making process, and principles of good practice in shared decision-making^18^ (discussing treatment options, communicating recommendation, discussing rationale for recommendation, revisiting options, eliciting and addressing questions or concerns, and codeveloping a plan).

### Outcomes

#### Primary Outcome

The 9-item Patient Health Questionnaire (PHQ-9) is a measure of depression symptoms, where each item is rated on a Likert scale ranging from 0 to 3 representing symptom frequency in the past 2 weeks, yielding an overall severity score ranging from 0 to 27.^19^ The cutoff of at least 10 is recommended to screen for clinically significant depression symptoms,^19^ and a change of at least 6 points is indicative of statistically reliable change.^25^ Patients in the IAPT program complete this measure on a session-by-session basis to monitor treatment response.^6^ Given that treatment duration is variable in routine care, the primary end point was defined at the time of each patient’s last attended treatment session.

The primary (preregistered) outcome of the study was the proportion of patients meeting criteria for reliable and clinically significant improvement (RCSI) in the PHQ-9 measure (posttreatment scores <10 and improved by ≥6 points). Reliable and clinically significant improvement is a clinically stringent and statistically conservative outcome that prioritizes full remission of symptoms,^26^ which is important in the context of stepped care, because patients who do not attain symptomatic remission have the opportunity to access further interventions to attain the best possible outcome. This outcome is consistent with the stratified care algorithm, which was specifically trained to calculate a prognosis (probability of RCSI) using this definition, and which was expected to result in better depression (PHQ-9) but not anxiety (Generalized Anxiety Disorder [GAD-7]) treatment outcomes based on prior evidence.^12^

#### Secondary Outcomes

We compared between-group differences in a range of secondary outcomes of interest. Reliable and clinically significant improvement status in the GAD-7^20^ was examined. Furthermore, IAPT services use an outcome definition termed *reliable recovery*, which is a stringent outcome definition that requires patients to have achieved RCSI in both the PHQ-9 and GAD-7 measures.^17^ Additional comparisons examined the proportions of patients who accessed low- vs high-intensity treatments, treatment duration (number of sessions), and treatment dropout (defined as unilateral discontinuation of treatment before the planned end of treatment). Adherence to the experimental intervention was measured by comparing the percentage of agreement and interrater reliability (κ statistic) in the stratified care model–recommended vs actual treatment selection decisions.

### Statistical Analysis

Data were analyzed from May 18, 2020, to October 13, 2021. All analyses followed intention-to-treat principles, and missing (n = 38) posttreatment PHQ-9 and/or GAD-7 scores were imputed with an expectation maximization method using baseline features as predictors. Data analysis was conducted in 5 steps. First, the proportion of patients with posttreatment remission (RCSI) of depression symptoms (PHQ-9) was compared between groups using logistic regression adjusted for baseline PHQ-9 scores. A mixed model (clustering by assessing clinician) was estimated first, showing no significant cluster effects (*P* = .11), so subsequent models applied a parsimonious logistic regression that improved goodness of fit (−2 log likelihood ratio test, 3262.44 [*df* = 1]; *P* < .001). A full output of the model-building process and goodness-of-fit indices can be found in eMethods 3 in Supplement 2. Second, logistic regressions were repeated in the subsamples of standard and complex cases. Third, these analyses were repeated using the anxiety (GAD-7) outcome measure and IAPT reliable recovery outcome definitions. Fourth, we compared additional secondary outcomes between groups using the χ^2^ and Mann-Whitney *U* tests. Fifth, an economic analysis evaluated the relative cost-effectiveness of stratified vs stepped care from a health services perspective using a cost-effectiveness acceptability curve to aid interpretation. Further details about sample size calculation and economic analyses are provided in eMethods 2 and 4, respectively, in Supplement 2. Two-sided *P* < .05 indicated statistical significance.

## Results

**Figure 1.**
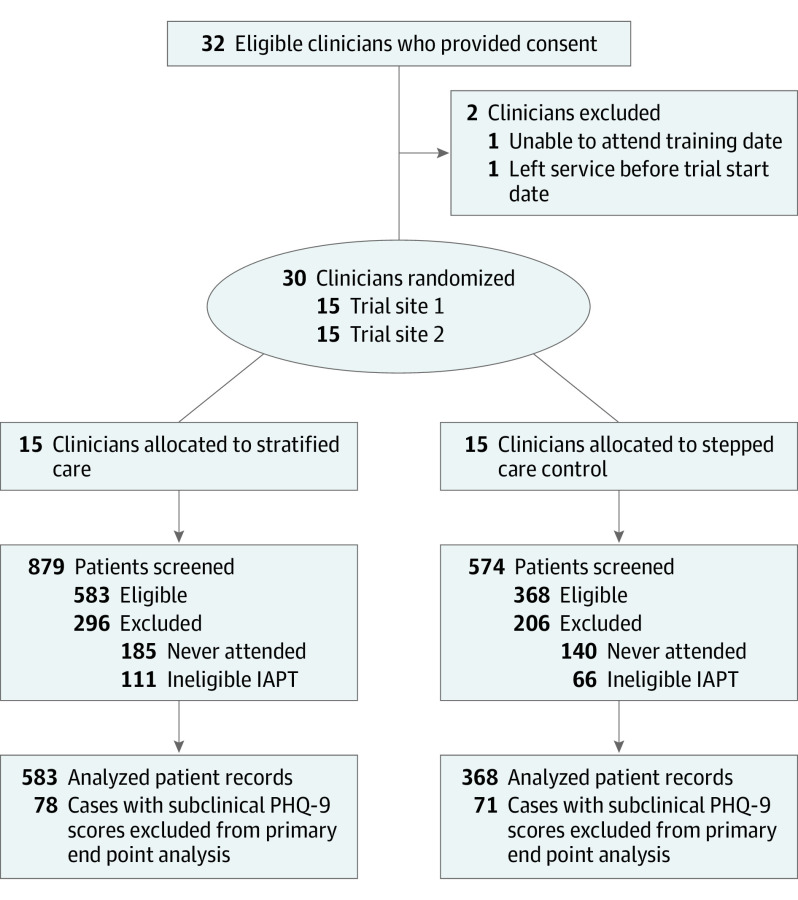
CONSORT Diagram IAPT indicates Improving Access to Psychological Therapies; PHQ-9, 9-item Patient Health Questionnaire.

The CONSORT diagram is presented in Figure 1. Thirty-two clinicians were recruited from July 5 to October 4, 2018; 2 withdrew before the start of the trial, and 30 were randomly allocated to the stratified care group (n = 15) or a stepped care control group (n = 15). Clinicians in both groups had the same qualifications and average weekly time availability for assessment tasks. Overall, 1453 patients were screened from August 20, 2018, to February 1, 2019, of whom 951 met eligibility criteria (583 in the stratified care group and 368 controls; 618 women [65.1%] and 332 men [34.9%] among 950 with data available; mean [SD] age, 38.27 [14.53] years). A small proportion of patients (149 [15.7%]) who did not score above the clinical cutoff in the PHQ-9 were excluded from the primary analysis (focusing on remission of clinically significant symptoms), but they were included in secondary analyses. Similarly, patients who did not score above the clinical cutoff in the GAD-7 measure (86 [9.0%]) were excluded from that specific analysis, but they were included in other secondary analyses. Detailed sample characteristics are presented in Table 1. In total, 225 of 951 patients (23.7%) were classified by the stratified care algorithm as complex cases.

**Table 1.** Patient Characteristics

Characteristic	Treatment group^a^		
	Full sample (n = 951)	Stratified care (n = 583)	Stepped care (n = 368)
Demographics			
Age, mean (SD), y	38.27 (14.53)	38.66 (14.61)	37.65 (14.41)
Sex			
Female	618/950 (65.1)	378/582 (64.9)	240/368 (65.2)
Male	332/950 (34.9)	204/582 (35.1)	128/368 (34.8)
Race and ethnicity^b^			
White	906/951 (95.3)	552/583 (94.7)	354/368 (96.2)
Other	45/951 (4.7)	31/583 (5.3)	14/368 (3.8)
Unemployed	187/951 (19.7)	131/583 (22.5)	56/368 (15.2)
Clinical features			
Primary diagnosis^c^			
Affective disorder	483/916 (52.7)	303/565 (53.6)	180/351 (51.3)
PTSD	27/916 (2.9)	16/565 (2.8)	11/351 (3.1)
OCD	14/916 (1.5)	6/565 (1.1)	8/351 (2.3)
Anxiety disorder	392/916 (42.8)	240/565 (42.5)	152/351 (43.3)
Prescribed pharmacotherapy	537/924 (58.1)	341/562 (60.7)	196/362 (54.1)
Comorbid long-term medical illnesses	182/932 (19.5)	100/574 (17.4)	82/358 (22.9)
Disability	103/921 (11.2)	61/572 (10.7)	42/349 (12.0)
SAPAS score, mean (SD)^d^	3.97 (1.43)	4.15 (1.44)	3.70 (1.37)
Complex cases	225/951 (23.7)	160/583 (27.4)	65/368 (17.7)
Baseline score, mean (SD)			
PHQ-9^e^	15.47 (5.86)	16.06 (5.69)	14.54 (6.01)
GAD-7^f^	14.21 (4.65)	14.57 (4.54)	13.64 (4.76)
WSAS^g^	20.33 (9.31)	21.24 (9.22)	18.96 (9.27)

Abbreviations: GAD-7, Generalized Anxiety Disorder questionnaire; OCD, obsessive-compulsive disorder; PHQ-9, 9-item Patient Health Questionnaire; PTSD, posttraumatic stress disorder; SAPAS, Standardised Assessment of Personality–Abbreviated Scale; WSAS, Work and Social Adjustment Scale.

^a^ Unless otherwise indicated, data are expressed as number/total number (%) of patients.

^b^ Information on race and ethnicity was self-reported by participants but aggregated in a binary variable (White British; other) by clinical services before data were shared with the research team. No other details about race and ethnicity were available to the research team.

^c^ Primary diagnosis was determined using a semistructured interview supplemented by validated case-finding measures for depression (PHQ-9) and anxiety disorders (GAD-7). Cases with missing data in each feature were excluded listwise.

^d^ Scores range from 0 to 8, with higher scores indicating more personality disorder traits.

^e^ Scores range from 0 to 27, with higher scores indicating more severe depression symptoms.

^f^ Scores range from 0 to 21, with higher scores indicating more severe anxiety symptoms.

^g^ Scores range from 0 to 40, with higher scores indicating greater impairment to work and social functioning.

Table 2 summarizes the results of primary and secondary outcomes. Overall, in the full sample, patients in the stratified care group had significantly better depression (PHQ-9) treatment outcomes (RCSI: 264 of 505 [52.3%] vs 134 of 297 [45.1%]; OR, 1.40 [95% CI, 1.04-1.87]; *P* = .03). Patients in the stratified care group were also significantly more likely to meet criteria for IAPT reliable recovery (276 of 573 [48.2%]) after treatment compared with patients in the stepped care group (152 of 348 [43.7%]; OR, 1.33 [95% CI, 1.01-1.75]; *P* = .04). Subgroup analyses indicated that between-group differences in depression outcomes were not significant in the subsample of complex cases (RCSI: 63 of 160 [39.4%] vs 22 of 65 [33.8%]; OR, 1.28 [95% CI, 0.70-2.35]; *P* = .42), but they were significant in the subsample of standard cases (RCSI: 201 of 345 [58.3%] vs 112 of 232 [48.3%]; OR, 1.50 [95% CI, 1.07-2.09]; *P* = .02). Between-group comparisons in the anxiety outcome measure were not statistically significant (eg, full-sample RCSI, 266 of 538 [49.4%] vs 151 of 327 [46.2%]; OR, 1.19 [95% CI, 0.90-1.57]; *P* = .22).

**Table 2.** Treatment Pathway and Outcomes

Characteristic	Treatment group^a^		Between-group comparisons^b^	*P* value
	Stratified care (n = 583)	Stepped care (n = 368)		
**Treatment pathway**				
LIT	251/583 (43.1)	261/368 (70.9)	χ^2^ = 70.51	<.001
HIT^c^	332/583 (56.9)	107/368 (29.1)		
Treatment sessions, mean (SD)	7.10 (5.31)	5.84 (4.15)	Mann-Whitney *U* test, 121106.00 (SE, 4098.98)	<.001
Treatment dropout	166/542 (30.6)	107/348 (30.7)	χ^2^ = 0.001	.97
Adherence to the stratified care model	523/583 (89.7)	233/368 (63.3)	χ^2^ = 96.41	<.001
κ Statistic	0.81	0.22	NA	NA
**Treatment outcomes**				
PHQ-9 depression RCSI				
Full sample	264/505 (52.3)	134/297 (45.1)	1.40 (1.04-1.87)	.03
Complex cases subsample	63/160 (39.4)	22/65 (33.8)	1.28 (0.70-2.35)	.42
Standard cases subsample	201/345 (58.3)	112/232 (48.3)	1.50 (1.07-2.09)	.02
GAD-7 anxiety RCSI				
Full sample	266/538 (49.4)	151/327 (46.2)	1.19 (0.90-1.57)	.22
Complex cases subsample	52/160 (32.5)	21/65 (32.3)	1.02 (0.55-1.89)	.96
Standard cases subsample	214/378 (56.6)	130/262 (49.6)	1.35 (0.98-1.85)	.07
IAPT reliable recovery, full sample^d^	276/573 (48.2)	152/348 (43.7)	1.33 (1.01-1.75)	.04

Abbreviations: GAD-7, Generalized Anxiety Disorder questionnaire; HIT, high-intensity treatments; IAPT, Improving Access to Psychological Therapies; LIT, low-intensity treatments; PHQ-9, 9-item Patient Health Questionnaire; RCSI, reliable and clinically significant improvement.

^a^ Unless otherwise indicated, data are expressed as number/total number (%) of patients.

^b^ Unless otherwise indicated, data are expressed as odds ratio (95% CI).

^c^ Of these, 46 (13.9%) had prior LIT in the stratified care group and 28 (7.6%) had prior LIT in the stepped care group.

^d^ Requires patients with case-level PHQ-9 and/or GAD-7 symptoms to have (1) attained statistically reliable improvement on case-level measures, (2) to have subclinical symptoms on both measures after treatment, and (3) to not have statistically reliable deterioration on any of these measures after treatment.

**Figure 2.**
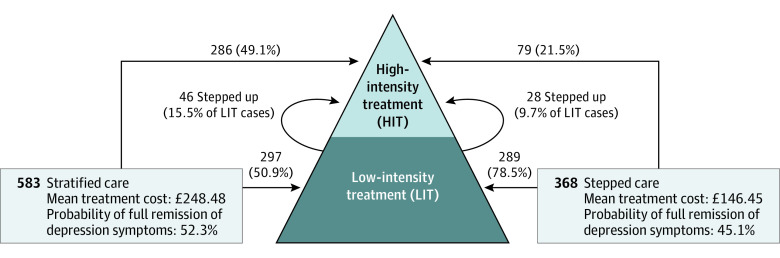
Treatment Pathways, Costs, and Outcomes in Stratified and Stepped Care To convert costs to US dollars, multiply pounds sterling by 1.338.

Stratified care was associated with a higher median number of treatment sessions (6 [IQR, 3-9]; range, 1-30) compared with stepped care (5 [IQR, 3-8]; range, 1-25) (Mann-Whitney *U* test, 121106.00 [SE, 4098.98]; *P* < .001). This is explained by the higher proportion of patients who accessed high-intensity interventions in stratified care (332 of 583 [56.9%] vs 107 of 368 [29.1%]; χ^2^ = 70.51; *P* < .001), because dropout rates were not significantly different (166 of 542 [30.6%] vs 107 of 348 [30.7%]; χ^2^ = 0.001; *P* = .97), but resulted in an approximately 7% increase in the probability of RCSI. Adherence to the stratified care model was high in the experimental group (κ = 0.81) and significantly different from the treatment selection decisions observed in the stepped care group, which had low concordance with the stratified care algorithm (κ = 0.22). As illustrated in Figure 2, the stratified care pathway allocated only half of patients (297 of 583 [50.9%]) to low-intensity treatments, whereas most of the patients (289 of 368 [78.5%]) were initially allocated to low-intensity treatments in the stepped care group.

**Figure 3.**
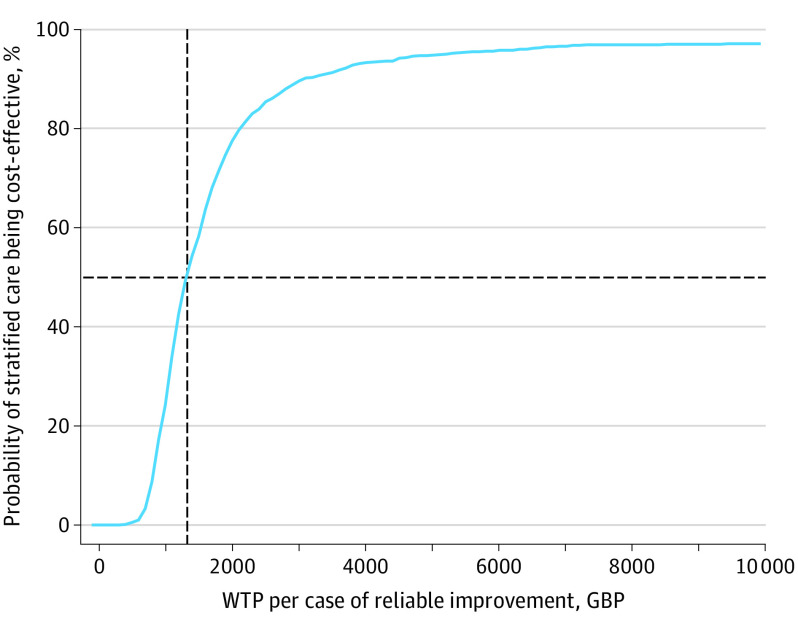
Cost-effectiveness Acceptability Curve Probability of stratified care being cost-effective (vs stepped care) is greater than 50% if the willingness-to-pay (WTP) threshold is greater than £1320 ($1766.31) per additional case of reliable improvement (dashed lines). GBP indicates pound sterling currency.

The estimated incremental cost of stratified care was £104.5 (95% CI, £67.5-£141.6) per patient ($139.83 [95% CI, $90.32-$189.48] per patient) (*P* < .001). The cost-effectiveness acceptability curve in Figure 3 shows that the probability of stratified care being cost-effective, compared with stepped care, is 50% when the willingness-to-pay threshold per additional case of reliable improvement is £1320 ($1766.31). The probability of stratified care being cost-effective increases to 80% and 90% for the willingness-to-pay values of £2100 ($2810.03) and £3050 ($4081.24), respectively. Further details of the economic analysis are available in eMethods 4 and eFigures 1 and 2 in Supplement 2.

## Discussion

A growing literature in the field of depression suggests that treatment outcomes could be improved through personalized treatment selection.^27^ The findings of this trial indicate that stratified care improves depression outcomes, albeit at an incremental cost per treatment. This improvement comes with no effect on dropout rates, despite the fact that significantly more patients in the stratified care group accessed high-intensity treatments, which have longer waiting lists. Dropout rates in the present study and across both trial arms (approximately 30%) were consistent with data from IAPT services.^7^ Furthermore, the stratified care model was feasible to implement and had a high adherence rate (κ= 0.81). Treatment selection decisions made in the stepped care group had nearly chance-level convergence with those in the stratified care algorithm (κ= 0.22), indicating that decision-making across these models is highly distinctive. Stratified care also increased the efficiency of initial assessments, because clinicians in the experimental group were able to assess a larger sample of patients in the same allotted weekly time, whereas decisions in the stepped care group were sometimes protracted by the need to consult with colleagues or supervisors about suitability for available treatments, which is commonplace in stepped care.^23^

As expected, the proportions of patients with full remission (RCSI) of depression and anxiety symptoms were higher in stratified care compared with stepped care, but differences were statistically significant only in the PHQ-9 measure. This is consistent with prior evidence suggesting that stratified care could improve remission rates in the PHQ-9 but not in the GAD-7 measure.^12^ A plausible explanation is that there was little difference in the treatment allocation of patients with anxiety disorders between stratified and stepped care, because both models refer patients with some conditions (eg, posttraumatic stress disorder) directly to high-intensity treatments in line with clinical guidelines.^1,17^ These results are consistent with prior evidence that IAPT services that have a higher proportion of patients accessing high-intensity treatments tend to have better treatment outcomes.^17,25^ Related to this point, a possible explanation for the observed effect may be owing to an absolute increase in the proportion of patients allocated to high-intensity treatment, rather than a strategic matching effect. Future implementation trials could examine whether the effect of stratified treatment selection varies across IAPT services, with variable proportions of patients allocated to high-intensity treatment.

Contrary to our expectations, only standard (ie, less complex) cases had significantly better outcomes in stratified care. In the present study, relatively few patients with the poorest expected prognosis were classified as complex cases (225 of 951 [23.7%]), and it may be that the observed trend toward better outcomes in stratified care for the complex cases could be diluted by the inclusion of some patients with chronic conditions that simply do not respond to interventions available in IAPT services. Previous research^28^ suggests that the presence of patients with chronic conditions in a clinical sample may obscure the differential treatment response in those with more treatable conditions. It is, of course, possible that stratified care does not work for complex cases as defined in this study, and future research should consider how to improve outcomes for those at the highest risk of poor treatment response.

### Limitations

The pragmatic trial design maximized feasibility, sample size, and external validity to the routine care context, but inevitably had some weaknesses in terms of internal validity. Outcomes were patient reported, and no formal diagnostic interviews or observer-rated outcomes were available. The sole reliance on patient-reported measures means that we cannot rule out or examine the potential influence of biases such as motivated responding and social desirability bias. Although the PHQ-9 and GAD-7 measures are relevant to the broad range of affective and anxiety symptoms reported by participants, a significant limitation is that disorder-specific measures were unavailable for conditions such as posttraumatic stress disorder and obsessive-compulsive disorder. In addition, most of the participants were White individuals, which limits the generalizability of these findings to other racial and ethnic minority groups. Furthermore, outcomes were defined at the last attended treatment session, and therefore the maintenance of these effects over a longer time frame could not be established. A further implication is that, on average, the final outcomes in the stratified care group were measured at a later time compared with those in stepped care, because more patients had lengthier high-intensity treatments in the experimental group. Thus, there are uncertainties related to the pragmatic design, and future studies could establish a fixed follow-up measurement schedule to understand short- and longer-term effects with greater precision. The economic analysis was limited to a comparative examination of acute-phase treatment costs, but wider outcomes such as quality-adjusted life-years and use of health services after the end of treatment remain unknown.

## Conclusions

Overall, the present findings indicate that stratified care is feasible to implement in routine IAPT services, improving the efficiency and precision of psychological assessments in a way that preserves shared decision-making. Implementation of stratified care resulted in better depression treatment outcomes albeit with an additional cost per treatment.

## Notes

Supplement 1.
Trial Protocol

Supplement 2. **eMethods 1.** Professional background of Psychological Well-being Practitioners **eMethods 2.** Sample Size Calculation **eMethods 3.** Preliminary Model Building and Examination of Cluster Effects **eMethods 4.** Economic Analysis **eFigure 1.** Cost-effectiveness Acceptability Curve, Showing the Probability of Stratified Care Being Cost-effective for a Range of Willingness-to-Pay Threshold for 1 Additional Case of Reliable Improvement **eFigure 2.** Cost-effectiveness Plane, Showing Incremental Difference in Cost and Probability of Reliable Improvement Between Stratified Care and Stepped Care Models
eReferences

Supplement 3.
Data Sharing Statement
