# Supplementary material for: Stratified Care vs Stepped Care for Depression: A Cluster Randomized Clinical Trial
Source: JAMA Psychiatry. 2021 Dec 8;79(2):1–9. doi: 10.1001/jamapsychiatry.2021.3539 (PMC8655665; doi:10.1001/jamapsychiatry.2021.3539)
Supplement: Supplement 1. — Trial Protocol [file jamapsychiatry-e213539-s001.pdf]

RESEARCH PROTOCOL
Pragmatic randomised controlled trial of a stratified care model for depression and anxiety

Short title of study	
StratCare Trial	
**Protocol version and date**	**Research Ethics Committee (REC) reference**
(v3) 22.02.19	18/WS/0114
**Sponsor organisation**	**Controlled trials registration number**
Lancashire Care NHS Foundation Trust	https://doi.org/10.1186/ISRCTN11106183

Research team

Jaime Delgadillo[1,5], Michael Barkham[1], Kieran Fleck[2], Charlotte Clay[2], Laura Baverstock[2], Lisa Woods[2], Zachary D Cohen[3], Robert J DeRubeis[3], Shehzad Ali[4], Victoria Adam[5]

1 – Clinical Psychology Unit, Department of Psychology, University of Sheffield, UK
2 –Lancashire Care NHS Foundation Trust, UK
3 – Department of Psychology, University of Pennsylvania, USA
4 – Centre for Health Economics and Department of Health Sciences, University of York, UK
5 – Rotherham, Doncaster and South Humber NHS Foundation Trust

Principal investigator contacts details

J. Delgadillo
Clinical Psychology Unit, Cathedral Court, Floor F, 1 Vicar Lane, Sheffield S1 1HD
Tel: 0114 222 6614; Email: j.delgadillo@sheffield.ac.uk

Contents of the study protocol	Pg.
1. Synopsis	2
2. Background and rationale	3
3. Objectives and research questions	3
4. Study design	4
4.1 Setting	4
4.2 Participants	4
4.3 Measures	5
4.4 Recruitment, study procedures and data collection	5
5. Data analysis	8
6. Ethical considerations	9
7. References	10

1. Synopsis of the study	
**Short study title**	StratCare Trial
**ISRCTN registration no.**	ISRCTN11106183
**Study Design**	Pragmatic, multi-site cluster randomised controlled trial
**Setting**	IAPT services in Lancashire Care NHS Trust and RDaSH NHS Trust
**Study Participants**	Clinicians that carry out routine assessments in an IAPT service, and patients that undergo assessments
**Primary Objective**	To evaluate the effectiveness of applying a Stratified Care treatment-matching model in routine psychological care (StratCare).
**Secondary Objectives**	▪ To compare anxiety symptoms, IAPT *reliable recovery* rates and treatment dropout rates between patients assessed in the StratCare condition, versus usual care control cases.<br>▪ To assess clinicians' adherence to the StratCare model.<br>▪ To assess the cost-effectiveness of stratified care, by comparison to usual stepped care
**Primary outcome(s)**	Patient-level depression (PHQ-9) symptom measure.
**Randomization and interventions**	Consenting therapists and patients will be randomly assigned to a StratCare group or usual care control group. StratCare therapists will be trained to use an algorithm to match patients to specific treatments available in the IAPT service.
**Planned Sample Size**	A minimum sample of 10 therapists and 760 cases is required.
**Data analysis method**	1. Trial data will be summarised using a CONSORT diagram and analyses will be based on *intention-to-treat* principles.<br>2. Patient-outcome data will be analysed using logistic regression. Post-treatment reliable and clinically significant improvement (RCSI) in depression symptoms will be the primary outcome, and will be compared between StratCare and usual care control groups.<br>3. Subgroup analyses will examine between-group differences in the subsamples of patients classified as *standard* and *complex* cases.<br>4. Secondary analyses will involve comparing between-group differences in anxiety symptoms, *reliable recovery* and dropout rates. We will also assess adherence to the StratCare model using Kappa statistics in the full sample.<br>5. An economic analysis will be conducted in the full sample using a cost-effectiveness acceptability curve (CEAC) to represent the probability of StratCare being cost-effective compared to usual care for different levels of willingness-to-pay for improvement in RCSI.
**Study Period**	18 months (1 year active study period, plus 6 months analyses and dissemination)

2. Background and rationale

Depression and anxiety are the most prevalent mental health problems and can become chronic and disabling for many people. Patients with depression and anxiety problems accessing the English National Health Service are commonly referred for psychological treatment in IAPT services (*Improving Access to Psychological Therapies*). IAPT services organise treatment in a stepped care model, where most patients tend to initially receive brief and low intensity interventions prior to accessing more intensive psychological therapies if required (Clark, 2011). Whilst this sequential and stepped care approach has been recommended as a cost-effective way to deliver psychological interventions (National Institute for Health and Care Excellence, 2011), it is evident that at least 25% of patients drop out shortly after starting treatment (Richards & Borglin, 2011) and dropout is associated with poor treatment outcomes (Delgadillo et al., 2014). Furthermore, recent studies in IAPT services indicate that patients with specific prognostic characteristics tend to be at higher risk of dropout and poor outcomes, particularly in low intensity interventions (Delgadillo, Moreea, & Lutz, 2016; Delgadillo, Huey, Bennett, & McMillan, 2017).

These observations have important clinical implications. Patients who drop out of low intensity interventions often do not have the opportunity to access high intensity interventions, as intended by the 'self-correcting' mechanism of stepped care (Bower & Gilbody, 2005). Furthermore, more complex cases that do not benefit from low intensity treatment and who do eventually access high intensity treatment appear to respond less well, compared to those who are initially 'matched' to high intensity treatment at the start of their care pathway (Delgadillo et al., 2017). Taken together, these preliminary findings suggest that applying 'stratified care' in which patients are matched to specific interventions may be a more efficient and effective way to recommend available treatments. The 'treatment-matching' hypothesis has recently garnered interest in psychiatry and clinical psychology, with the emergence of a wave of studies that demonstrate how data-driven predictive models can help to identify subgroups of cases that respond well to certain treatments and not others (see review by Cohen and DeRubeis, 2018). Such studies indicate that individual-patient data can be used to determine which patients respond best to alternative pharmacological treatments (e.g., Checkroud et al., 2016), psychotherapies for depression (e.g., DeRubeis et al., 2014; Huibers et al., 2015), or treatment options within a stepped care model (e.g., Delgadillo et al., 2016, 2017; Lorenzo-Luaces, DeRubeis, van Straten, & Tiemens, 2017). Despite this conceptual and methodological progress, most studies on personalized treatment-matching in mental health are based on retrospective data from trials and naturalistic clinical samples. To date, there are no published *prospective or experimental* tests of the stratified care model in psychological services.

Accordingly, this study aims to further our understanding of stratified mental health care by experimentally testing the 'treatment-matching' hypothesis, using a prospective randomised controlled trial design.

3. Objectives and Hypotheses

3.1. Primary Objective

To evaluate the effectiveness of a stratified care model of treatment selection (StratCare), applied in IAPT psychological treatment services. Effectiveness will be defined by comparing the proportions of cases with full remission of depression symptoms in a stratified care versus a stepped care pathway.

3.2. Secondary Objectives

- To examine between-group (StratCare vs. stepped care) differences in treatment outcomes within the subgroups of patients classified as *standard* and *complex* cases.

- To assess if StratCare differentially impacts on anxiety symptom changes (secondary outcome measure).
- To compare IAPT *reliable recovery* and dropout rates between patients who are assigned to treatment using StratCare versus usual stepped care.
- To compare dropout rates between patients who are assigned to treatment using StratCare versus usual stepped care.
- To assess clinicians' adherence to the StratCare treatment-selection model.
- To assess the cost-effectiveness of stratified care, by comparison to usual stepped care.

3.3. Hypotheses

- The StratCare group will have higher rates of reliable and clinically significant improvement (RCSI) in depression symptoms, compared to those in the stepped care group.
- *Complex cases* in the StratCare group will have higher rates of reliable and clinically significant improvement (RCSI) in depression symptoms, compared to those in the control group.
- Patients whose treatment assignment is guided by StratCare will have significantly lower treatment dropout rates compared to cases in the control group.
- An index of adherence to the StratCare treatment selection model will be significantly higher in therapists assigned to the StratCare group, compared to those assigned to the control group.

4. Study design

This will be a multi-site, pragmatic, cluster randomised controlled trial. Patients' outcome data will be nested within therapists, and randomization will be applied at therapist (cluster) level. Psychological therapists will be randomly allocated to StratCare vs. usual stepped care (control) groups using electronic randomisation software. We will collect clinical records for all consenting patients assessed by both groups of therapists during a one-year study period.

A cluster design has been adopted to minimise the chance of contamination which may occur if randomization is applied at patient-level, since it is possible that a clinician who is familiar with the StratCare model may apply this in control group cases. This is particularly relevant in an IAPT service because patients' clinical and demographic data is routinely available to all clinicians.

4.1. Setting

The study will be conducted in Lancashire Care NHS Trust and Rotherham Doncaster and South Humber NHS Foundation Trust. These organisations manage multiple IAPT psychological services. IAPT is a national programme offering evidence-based psychological interventions for depression and anxiety related conditions (Clark, 2011). Treatment is organised in a stepped care model, which enables access to brief (typically ≤ 8 sessions) and low intensity therapies initially, and offers more intensive therapies (up to 20 sessions) for patients who do not improve at the earlier steps of care. Patients receiving psychological therapies in IAPT are typically seen in primary care clinics and other community based venues. Trial participants will therefore be qualified therapists that routinely carry out initial assessments in routine IAPT services.

4.2. Study Participants

Inclusion criteria

- Consenting psychological wellbeing practitioners and psychotherapists that carry out routine assessments in an IAPT service.
- Therapists who are employed by a participating IAPT service on a permanent contract, or temporary staff who have a contract that is at least as long as the expected timescale for the project (1 year).
- All consenting patients who are assessed by participating therapists, who are deemed eligible for treatment in IAPT, and who attend at least one post-assessment therapy session.

Exclusion criteria

- Therapists whose contract is shorter than the expected timescale for the study (1 year).
- Therapists currently in training, since they are not yet fully qualified to carry out routine assessments.
- Patients who are assessed as ineligible for treatment in IAPT (e.g., those who are signposted to other services), or eligible patients who never attend any therapy sessions after an initial assessment contact.

4.3. Measures

IAPT services monitor clinical outcomes by asking patients to complete brief symptom questionnaires on a session-to-session basis, which is standard practice (Clark, 2011). These questionnaires are collected by therapists and results are routinely entered into an electronic case record system.

Primary outcome measure

The PHQ-9 is a brief measure of depression symptoms (Kroenke et al., 2001). Each of the nine items is scored on a 0–3 scale and these are summed to give an overall severity rating (range 0–27). The PHQ-9 has been extensively validated in primary care populations (Kroenke et al., 2010), with adequate sensitivity (88%) and specificity (88%) estimates for the detection of major depressive disorder using a cut-off score ≥10.

Secondary measures

GAD-7 is a seven item measure of common anxiety symptoms (Spitzer et al., 2006). Each item is scored on a 0–3 scale and these are summed to give an overall severity rating (range 0–21). The GAD-7 has been found to be a reliable screening tool for anxiety disorders such as generalized anxiety, social phobia, post-traumatic stress and panic disorder (Kroenke et al., 2007). A cut-off score ≥8 in this measure has been shown to detect an anxiety disorder with adequate sensitivity (77%) and specificity (82%).

The WSAS (Mundt et al., 2002) questionnaire assesses the impact of mental health problems on 5 life domains (work, home management, social life, leisure activities, family and relationships) using Likert scales ranging from 0 (no impairment) to 8 (severe impairment). Scores across all 5 domains are summed to derive an overall score of functional impairment.

The Standardized Assessment of Personality–Abbreviated Scale (SAPAS) is an eight-item questionnaire developed to screen for the likely presence of a personality disorder (Moran et al., 2003).

Each question prompts respondents to endorse specific personality traits (yes/no), yielding a total score between 0 and 8 where a cut-off >3 is indicative of cases with a high probability of diagnosable personality disorders. The WSAS and SAPAS will be gathered at the time of initial assessments.

In addition to the above clinical outcome measures, we will collect anonymized data for patients treated by the participating therapists, and which is gathered in routine practice by IAPT services. These data will include demographics (e.g. age, gender, ethnicity, employment status, index of multiple deprivation, self-reported disabilities) and clinical care data (e.g. diagnoses, number of therapy sessions, types of treatments offered, reason for discharge, last step accessed in stepped care system).

We will also collect data on patients' perceived therapeutic alliance and outcome expectancy for treatment. The patient version of the Working Alliance Inventory Short-Form (WAI-SF; Tracy & Koktovic, 1989) is a 12-item measure of therapeutic alliance between patient and practitioner. Therapeutic alliance is a well-established predictor of therapeutic outcomes (Flückiger, Del Re, Wampold & Horvath, 2018), particularly patient ratings of the alliance (Summers & Barber, 2003). The WAI is one of the most widely used and reliable measures for assessing the alliance (Hatcher & Gillaspy, 2006). Three components of the alliance are measured: agreement on tasks, agreement on goals and the patient-therapist bond (Tracey & Koktovic, 1989). Each item is rated on a seven-point scale (1 = never to 7 = always) and higher overall scores indicate a stronger therapeutic alliance.

Patients' expectation of therapeutic outcome will be measured by asking "*At this point in time, how confident are you that this kind of treatment will work for you on a scale of 0 (not at all) to 10 (definitely)?*" There is a reliable relationship between positive expectancy for therapy and better treatment outcomes (Constantino, Visla, Coyne & Boswell, 2018). Scores of 5 or below on this scale are indicative of low expectancy and therefore higher risk of poorer treatment outcomes (Delgadillo, Moreea & Lutz, 2016).

The WAI-SF and outcome expectancy measure will be completed following the third session in each course of therapy a patient goes through. This is in line with evidence that patients' outcome expectancy measured at this point in the course of treatment is associated with clinical outcomes (Constantino, Arnkoff, Glass, Ametrano & Smith, 2011).

4.4. Recruitment, study procedures and data collection

Therapist recruitment process

- Clinical collaborators at each participating IAPT team will email copies of the participant information sheet (Appendix 1) and consent form (Appendix 2) to all therapists in their service.
- Clinical collaborators may also promote the study at their local team meetings (or delegate this task to a colleague).
- Therapists will have at least 1 week to consider their participation and to contact the research team to clarify questions if necessary prior to the deadline for submitting consent forms. They will be asked to submit signed consent forms directly to the research team at the University of Sheffield – either via email (scanned copy of signed consent form) or via post. Local Clinical collaborators will not process the consent forms, which will minimise administrative burden but also minimise the potential for selection biases or undue pressure (e.g. therapists feeling that they must consent to participate if their manager is receiving consent forms).
- Consenting therapists will be randomly allocated to a StratCare group, or a usual care control group. Allocation will be carried out by the research team, using randomisation software, and will be communicated directly to study participants via email.
- The research team based at Sheffield will inform local Clinical collaborators when their local recruitment target has been met, providing a list of consenting participants after the randomisation process has been concluded. This will ensure Clinical collaborators are able to make contact with their relevant colleagues for the purpose of organising local training events.

Patient recruitment process

- Participating therapists will use a brief and standardised script to obtain verbal consent from patients who they assess in routine care. The script is described in Appendix 3. The recruitment script will be read to all patients assessed by participating therapists, at the start of the assessment contact. If patients provide consent, the clinician will record their anonymised assessment information in a secure and confidential patient records system which is used in routine care. Participating therapists will also keep a spreadsheet where they also record how many cases refuse to consent to the study, so that we ensure that their clinical data is not included in the study dataset.
- All patients will have immediate access to a participant information sheet online (see Appendix 4), which will reduce postal costs and delays in receiving this information via post. This clearly explains how participants' anonymised information will be used for research purposes, and how they can withdraw from the study or make complaints if necessary.

Organisation of training event

- The research team will liaise with local Clinical collaborators to organise a training day which will be accessed by all participating therapists. Training events will be run prior to the start of the study, following a standard training agenda and materials. Participating therapists will have at least 2

weeks notice about the training date, to ensure they are able to make necessary arrangements to attend.

The StratCare treatment-matching model

- Therapists assigned to the StratCare group will be trained to use a clinical decision-making tool called the StratCare App.
- The StratCare App is computer programme that prompts therapists to enter specific information about each patient that is being assessed. This includes fully anonymised information including: PHQ-9 score, GAD-7 score, WSAS score, employment status, ethnicity, SAPAS personality traits. The App has a built-in algorithm that weighs up all of these variables to classify a case into one of two categories: *standard* or *complex case*. *Complex cases* are defined as those who have a combination of multiple features that are predictive of poor response to therapy. This algorithm has been derived from a study which demonstrated that *complex cases* have better treatment outcomes if they are matched to HIT (Delgadillo et al., 2017).
- Using the StratCare App, therapists will make treatment recommendations and discuss these with patients who they assess in routine care, during the study period. Therapists will be trained to explain their recommendation to patients in a way that enables shared decision-making and which also considers patients' preferences.

Data collection and safeguarding procedures

- The research team will work with a data manager at the clinical services to download a fully anonymized and aggregated dataset, excluding data from patients that did not provide consent. The dataset will include patient-level clinical data nested within therapist caseloads (which will be linked to a unique participant ID). The dataset will include anonymized data for up to 2 years prior to the start of the study – for the full treatment pathway including all clinical cases. This trial-within-cohort (TWIC) design will enable us to examine differences in assessment and treatment allocation practices before and after introducing the StratCare model. The dataset will assign anonymized identifiers to each case, which makes it impossible to personally identify any patients.
- Fully encrypted data will be transferred from the clinical service to the research team using a secure file transfer protocol (FTP). The dataset will be stored in a secure University network drive, only accessible to members of the research team. This will ensure the security and adequate storage of research data, consistent with NHS and academic codes of information governance and data protection.
- All analyses will be carried out at a University site, and data will be held in a restricted-access drive. The study dataset will be held at the University for a minimum of 5 years after the conclusion of the study.
- Participating therapists will be contacted by email to request that they complete an anonymised electronic survey gathering basic information for descriptive purposes (e.g., age, gender, years/months of experience carrying out mental health assessments, qualifications, etc.).
- Patients will be asked to complete the WAI-SF and outcome expectancy measure following their third session in therapy. Consenting patients' progress through treatment will be monitored via patient record systems that are used in routine care using participants' IDs from the dataset. A secure record of the expected date a patient will reach session three will be kept. Once the third session has been completed, patients will receive a text message asking them to complete an online survey that contains the WAI-SF and expectancy measure. If the patient's record does not contain a mobile phone number, researchers will call the participant on any other telephone number provided. A group of 10 current IAPT patients were asked their preferred method of contact, should we require to contact them during treatment, 8 patient expressed they would prefer a text message, and the remaining 2, who did not have text message facilities, stated they would be happy to be contacted via telephone. Patients will be assigned an ID number which will correspond with their unique, anonymous identifier allocated in the dataset and asked to enter this at the beginning of the survey. All data from this online questionnaire will be stored securely and confidentially. If

following the typical stepped-care approach to therapy, patients will complete this questionnaire after their third session at both step 2 and step 3 treatment levels.

5. Data analysis

5.1. Sample size calculation

A sample size calculation was performed using the method described by Fleiss, Levin, and Paik (2013), where the primary outcome is binary. The calculation was informed by the effect sizes (odds ratio and event base rates) described by Delgadillo et al. (2017). The following parameters were used for the sample size calculation: We expect that approximately 30% of cases assessed in routine care are likely to be classified as *complex cases* (which is the smallest expected subsample of interest, and therefore a useful guide to ensure the trial is powered to undertake subgroup analyses). Based on an expected Odds Ratio = 2.23, P_1 = 0.50, P_0 = 0.31, and risk ratio (P_1 / P_0) = 1.61; N = 113 per group would be required to detect a P_1 = 0.50 with 80% power. Considering the expected base rate of *complex cases*, we estimate that approximately 760 cases need to be assessed in routine care to identify 226 (113*2) *complex cases*.

There is no precedent for this type of trial in this setting, so it is not known if clustering effects are relevant for clinical assessments, nor do we have any prior information to calculate an intra-cluster correlation coefficient. Given the novel and pragmatic nature of this study, we have therefore followed conventional sample size calculation methods, and we will simply control for clustering using multilevel-modelling as explained below (if the random effect for the cluster level is significant and improves model-fit). This will enable us to assess if clustering effects are relevant or not in this context and given this specific outcome of interest.

Overall, we will aim to recruit a minimum of 10 therapists that carry out routine assessments in IAPT services. Between them, we expect that they will assess at least 760 during a 1-year study period, which would require each therapist to assess 2 cases per week on average.

5.2. Primary analysis

Patient-level clinical outcome data will be analysed using logistic regression. The primary outcome will be defined as reliable and clinically significant improvement (RCSI) in depression symptoms (PHQ-9) after treatment, based on the method described by Jacobson and Truax (1991). A 2-level multilevel logistic regression model will be applied initially, with patients nested within therapists that conducted initial assessments, and post-treatment RCSI in depression (PHQ-9) symptoms as the dependent variable. Group (StratCare vs. usual care) will be entered as a fixed effect, along with baseline PHQ-9 as a covariate. This method will enable us to determine whether StratCare is associated with a greater treatment effect (depression symptom remission) by comparison to usual care, and will be run in the full sample. The two-level model will account for the nested structure of the data, as appropriate within a cluster trial design. If the therapist-level random intercept is not statistically significant, a single-level parsimonious multivariate regression model will be estimated as the main analysis if it offers better goodness-of-fit to the data. Adjusted odds ratios and confidence intervals will be reported as the primary effect sizes.

Between-group comparisons in post-treatment PHQ-9 means will not be computed, because this is an aggregated (group-level) metric that is unsuitable to investigate patterns of differential response to treatment at the individual-level (patients). We do not expect to find between-group differences at an aggregated (mean) level, but we do expect to find differences in a binary (RCSI) outcome metric, which better quantifies outcomes of individuals, and which was the main outcome definition used to train the treatment selection algorithm used in the StratCare group.

5.3. Secondary analyses

The above modelling strategy will be repeated in the subsamples of patients classified as *standard* and *complex cases*. Furthermore, the rate of remission (RCSI) in GAD-7 anxiety symptoms will

be examined in the target sample of *complex cases* and in the full sample. The same method will be used to compare IAPT *reliable recovery* rates between groups, which is an outcome definition that combines RCSI status in both the PHQ-9 and the GAD-7 measures.

We will also compare dropout (versus treatment completion) rates between groups. For both StratCare and control groups, we will estimate the proportions of participants who had RCSI post-treatment, and who completed/dropped out of treatment. Treatment attendance, completion and dropout rates will be presented diagrammatically and based on CONSORT guidelines.

We will examine adherence to the StratCare model using the full study sample by calculating Kappa, which is a statistical measure of agreement between therapists' observed treatment recommendations and the recommendations that are output by the StratCare algorithm (see Delgadillo et al., 2017).

5.4. Economic evaluation

An economic evaluation will be conducted to evaluate the cost-effectiveness of StratCare versus usual care, in terms of incremental cost per unit of effectiveness outcome. The analysis will be conducted from the NHS perspective. Direct treatment cost to deliver therapy sessions at low and high intensities will be used in the analysis. This will be estimated by multiplying each patient's total contact time with trial therapists by the standard cost per hour at the relevant pay rates*, based on the Unit Costs of Health and Social Care 2016 (Curtis, 2016). The effectiveness outcome for the cost-effectiveness analysis will be in line with the clinical analysis, i.e. RCSI defined in terms of PHQ-9 outcome (primary analysis).

The statistical analysis will compare mean costs and outcomes in the StratCare and usual care arms. Multilevel regression analysis will be used to control for differences in baseline severity of symptoms, and to account for the cluster trial design (patients within therapists). Two regression models will be specified, one for the cost and other one for RCSI. The coefficient on the StratCare variable will represent the incremental difference in cost and RCSI. The incremental cost-effectiveness ratio (ICER) will be estimated as the ratio of the coefficients on StratCare in the cost and outcome regressions. Uncertainty in ICER will be estimated by bootstrapping the regression models. The bootstrap estimates will be presented on a cost-effectiveness plane. Finally, the results will be presented as a cost-effectiveness acceptability curve (CEAC) (Fenwick 2001) to represent the probability of StratCare being cost-effective compared to usual care for different levels of willingness-to-pay for improvement in RCSI. All economic analyses will be carried out using the full sample.

* The standard NHS cost per working hour estimates in pg. 137 were used for these calculations. Low intensity sessions (0.5 hour) were costed at Band 5; high intensity counselling sessions (1 hour) were costed at Band 6; high intensity CBT sessions (1 hour) were costed at Band 7.

6. Ethical considerations

6.1. Considerations about informed consent

Given the nature of the StratCare intervention, we consider therapists to be the primary study participants. Patients will not be required to do anything different to treatment as usual. The only difference will be that therapists in the StratCare group will have access to a decision-making tool (StratCare App) that will guide their treatment recommendations, in a way that we expect will improve treatment outcomes. Assessing new patients and making a treatment recommendation are routine procedures in psychological care. However, we expect that the StratCare model will support therapists to make treatment recommendations in a more consistent and effective way. We have previously obtained ethical approval for other studies of clinical decision-making tools in which we recruited therapists as primary participants (REC reference: 15/NW/0675; REC reference: 15/LO/2200). This trial will follow the same principles, recruitment and training procedures, and –as shown by our previous studies of clinical decision tools– we expect this to be feasible, acceptable and without any major ethical challenges.

In order to obtain informed consent from therapists in line with good practice guidelines, we will take the following steps:

- Planned local team meeting visits will enable potential participants with an opportunity to ask questions, raise concerns and discuss any aspects of the study that they wish to clarify. Therapists will also be invited to email or call a member of the research team if they have any further thoughts or questions after team meetings.
- Potential participants will have 1 week to consider their participation, so that they do not feel unduly pressured to consent at the time of team meeting visits.
- Potential participants will be advised of their right to withdraw from the study at any stage and the right to request their data to be deleted from the study dataset.

We will also be collecting fully anonymous patient-level data described in section 4.4. We consider that our proposed method for aggregating and analysing fully anonymized patient data is congruent with the NHS information governance policy and good practice guidelines. We will also obtain verbal consent from patients and they will have immediate access to information on how to withdraw their data from the study if they wish to do so (the full consent process for patients is described in the Appendix titled: StratCare_Patients_Consent_process_v1).

7. References

Bower, P., & Gilbody, S. (2005). Stepped care in psychological therapies: access, effectiveness and efficiency. *British Journal of Psychiatry, 186*, 11-17.

Chekroud, A.M., Zotti, R.J., Shehzad, Z., Gueorguieva, R., Johnson, M.K., Trivedi, M.H., Cannon, T.D., Krystal, J.H. and Corlett, P.R., 2016. Cross-trial prediction of treatment outcome in depression: a machine learning approach. *The Lancet Psychiatry, 3*, 243-250.

Clark, D. M. (2011). Implementing NICE guidelines for the psychological treatment of depression and anxiety disorders: The IAPT experience. *International Review of Psychiatry, 23*, 318–327.

Curtis L, Burns A. *Unit Costs of Health and Social Care 2016*. Personal Social Services Research Unit, 2016.

Delgadillo, J., McMillan, D., Lucock, M., Leach, C., Ali, S. and Gilbody, S. (2014). Early changes, attrition and dose-response in low intensity psychological interventions. *British Journal of Clinical Psychology*, 53, 114–130.

Delgadillo, J., Moreea, O., & Lutz, W. (2016). Different people respond differently to therapy: A demonstration using patient profiling and risk stratification. *Behaviour Research and Therapy, 79*, 15–22.

Delgadillo, J., Huey, D., Bennett, H., McMillan, D. (2017). Case Complexity as a Guide for Psychological Treatment Selection. *Journal of Consulting and Clinical Psychology, 85*, 835–853.

DeRubeis, R. J., Cohen, Z. D., Forand, N. R., Fournier, J. C., Gelfand, L. A., & Lorenzo-Luaces, L. (2014). The personalized advantage index: Translating research on prediction into individualized treatment recommendations. A demonstration. *PLoS One, 9*(1), e83875. doi: 10.1371/journal.pone.0083875

Cohen, Z.D., DeRubeis R.J. (2018). Treatment Selection in Depression. *Annual Review of Clinical Psychology, 14,* 15.1–15.28.

Constantino, M. J., Vîslă, A., Coyne, A. E., & Boswell, J. F. (2018). A meta-analysis of the association between patients' early treatment outcome expectation and their posttreatment outcomes. *Psychotherapy*, *55*(4), 473.

Constantino, M. J., Arnkoff, D. B., Glass, C. R., Ametrano, R. M., & Smith, J. Z. (2011). Expectations. *Journal of clinical psychology*, *67*(2), 184-192.

Delgadillo, J., Moreea, O., & Lutz, W. (2016). Different people respond differently to therapy: A demonstration using patient profiling and risk stratification. *Behaviour Research and Therapy*, *79*, 15-22.

Fenwick E, Claxton K, Sculpher M. (2001). Representing uncertainty: the role of cost-effectiveness acceptability curves. *Health Economics, 10*(8), 779-87.

Fleiss, J. L., Levin, B., & Paik, M. C. (2013). *Statistical methods for rates and proportions*. John Wiley & Sons.

Flückiger, C., Del Re, A. C., Wampold, B. E., & Horvath, A. O. (2018). The alliance in adult psychotherapy: A meta-analytic synthesis. *Psychotherapy*. Advance online publication.

Hatcher, R. L., & Gillaspy, J. A. (2006). Development and validation of a revised short version of the Working Alliance Inventory. *Psychotherapy Research*, *16*(1), 12-25.

Huibers, M.J.H., Cohen, Z.D., Lemmens, L.H.J.M., Arntz, A., Peeters, F.P.M.L., Cuijpers, P., DeRubeis, R.J. (2015). Predicting Optimal Outcomes in Cognitive Therapy or Interpersonal Psychotherapy for Depressed Individuals Using the Personalized Advantage Index Approach. *PLoS One, 10*(11), e0140771. doi: 10.1371/journal.pone.0140771.

IAPT programme. (2011). The IAPT Data Handbook: Guidance on recording and monitoring outcomes to support local evidence-based practice version 2.0 (March 2011). Crown copyright 2010.

Jacobson, N. S. and Truax, P. (1991). Clinical significance: a statistical approach to defining meaningful change in psychotherapy research. *Journal of Consulting and Clinical Psychology*, 59, 12–19.

Kroenke, K., Spitzer, R. L., & Williams, J. B. W. (2001). The PHQ-9: Validity of a brief depression severity measure. *Journal of General Internal Medicine*, 16, 606–613.

Kroenke, K., Spitzer, R. L., Williams, J. B. W., Monahan, P. O., & Löwe, B. (2007). Anxiety disorders in primary care: Prevalence, impairment, comorbidity, and detection. *Annals of Internal Medicine*, 146, 317–325.

Kroenke, K., Spitzer, R. L., Williams, J. B. W., & Löwe, B. (2010). The Patient Health Questionnaire Somatic, Anxiety, and Depressive Symptom Scales: A systematic review. *General Hospital Psychiatry*, 32, 345–359.

Lorenzo-Luaces, L., DeRubeis, R. J., van Straten, A., & Tiemens, B. (2017). A prognostic index (PI) as a moderator of outcomes in the treatment of depression: A proof of concept combining multiple variables to inform risk-stratified stepped care models. *Journal of Affective Disorders, 213*, 78-85.

Moran, P., Leese, M., Lee, T., Walters, P., Thornicroft, G., & Mann, A. (2003). Standardised Assessment of Personality–Abbreviated Scale (SAPAS): Preliminary validation of a brief screen for personality disorder. *British Journal of Psychiatry, 183*, 228–232.

Mundt, J. C., Mark, I. M., Shear, M. K., Griest, J. M. (2002). The Work and Social Adjustment Scale: a simple measure of impairment in functioning. *British Journal of Psychiatry*, 180, 461-464.

National Institute for Health and Care Excellence (2011). *Common mental health disorders: Identification and pathways to care* [CG123]. London: National Institute for Health and Care Excellence. Retrieved from: http://www.nice.org.uk/guidance/CG123.

Richards, D., & Borglin, G. (2011). Implementation of psychological therapies for anxiety and depression in routine practice: Two year prospective cohort study. *Journal of Affective Disorders, 133*, 51-60.

Spitzer, R., Kroenke, K., Williams, J. B. W., & Lowe, B. (2006). A brief measure for assessing generalized anxiety disorder: The GAD-7. *Archives of Internal Medicine*, 66, 1092–1097.

Summers, R. F., & Barber, J. P. (2003). Therapeutic alliance as a measurable psychotherapy skill. *Academic Psychiatry*, *27*(3), 160-165.

Tracey, T. J., & Kokotovic, A. M. (1989). Factor structure of the working alliance inventory. *Psychological Assessment: A journal of consulting and clinical psychology*, *1*(3), 207.
