# Supplementary material for: Stratified Care vs Stepped Care for Depression: A Cluster Randomized Clinical Trial
Source: JAMA Psychiatry. 2021 Dec 8;79(2):1–9. doi: 10.1001/jamapsychiatry.2021.3539 (PMC8655665; doi:10.1001/jamapsychiatry.2021.3539)
Supplement: Supplement 2. — eMethods 1. Professional background of Psychological Well-being Practitioners eMethods 2. Sample Size Calculation eMethods 3. Preliminary Model Building and Examination of Cluster Effects eMethods 4. Economic Analysis eFigure 1. Cost-effectiveness Acceptability Curve, Showing the Probability of Stratified Care Being Cost-effective for a Range of Willingness-to-Pay Threshold for 1 Additional Case of Reliable Improvement eFigure 2. Cost-effectiveness Plane, Showing Incremental Difference in Cost and Probability of Reliable Improvement Between Stratified Care and Stepped Care Models eReferences [file jamapsychiatry-e213539-s002.pdf]

Supplementary Online Content

Delgadillo J, Ali S, Fleck K, et al. Stratified care vs stepped care for depression: a cluster randomized clinical trial. *JAMA Psychiatry*. Published online December 8, 2021. doi:10.1001/jamapsychiatry.2021.3539

eMethods 1. Professional background of Psychological Well-being Practitioners

eMethods 2. Sample Size Calculation

eMethods 3. Preliminary Model Building and Examination of Cluster Effects

eMethods 4. Economic Analysis

eFigure 1. Cost-effectiveness Acceptability Curve, Showing the Probability of Stratified Care Being Cost-effective for a Range of Willingness-to-Pay Threshold for 1 Additional Case of Reliable Improvement

eFigure 2. Cost-effectiveness Plane, Showing Incremental Difference in Cost and Probability of Reliable Improvement Between Stratified Care and Stepped Care Models

eReferences

This supplementary material has been provided by the authors to give readers additional information about their work.

eMethods 1. Professional background of Psychological Well-being Practitioners

The therapists that participated in this study and who conducted initial assessments were qualified Psychological Wellbeing Practitioners (PWPs) who were qualified with a nationally recognised *Postgraduate Certificate in Low Intensity Psychological Interventions*. This is a professional qualification in the United Kingdom, achieved through a one-year university training course which involves training in CBT theory, clinical skills training to deliver guided self-help, and supervised clinical practice. The course follows a standard national curriculum that is adhered to by the various universities offering this training, and requires trainees to be employed by a psychological service that is part of the national *Improving Access to Psychological Therapies* (IAPT) programme. Qualified PWPs carry out initial assessments to screen for common mental disorders and to determine suitability for treatment in IAPT services following national clinical guidelines. They also deliver brief (≤8 sessions), self-help oriented, CBT-based, structured and protocol-driven interventions endorsed by national guidelines for the treatment of depression, generalised anxiety disorder, specific phobias, panic disorder and other mild-to-moderate common mental health problems. These interventions are delivered through a variety of formats, including as individual guided self-help, group-based psychoeducation, telephone and internet-enabled interventions.

eMethods 2. Sample Size Calculation

This sample size calculation was estimated a priori, and pre-registered with the Health Research Authority in England (REF: 18/WS/0114) prior to the start of recruitment.

A sample size calculation was performed using the method described by Fleiss, Levin, and Paik (2013), where the primary outcome is binary. The calculation was informed by the effect sizes (odds ratio and event base rates) described by Delgadillo et al. (2017). The following parameters were used for the sample size calculation: We expect that approximately 30% of cases assessed in routine care are likely to be classified as *complex cases* (which is the smallest expected subsample of interest, and therefore a useful guide to ensure the trial is powered to undertake subgroup analyses). Based on an expected Odds Ratio = 2.23, $P_1 = 0.50$, $P_0 = 0.31$, and risk ratio (P_1 / P_0) = 1.61; N = 113 per group would be required to detect a $P_1 = 0.50$ with 80% power. Considering the expected base rate of *complex cases*, we estimate that approximately 760 cases need to be assessed in routine care to identify 226 (113*2) *complex cases*.

The observation that clinical outcomes vary between different therapists is well established in the psychotherapy literature, and these *therapist effects* have been estimated to account for approximately 5% of variance according to meta-analytic reviews (Baldwin & Imel, 2013). However, there is no prior evidence that clinical outcomes are influenced in this way by clinicians that assess patients before they start therapy, especially in clinical contexts where the *assessing clinician* is different to the clinician that actually delivers the therapy. In the StratCare Trial, patients were clustered within *assessing clinicians*, who were randomly assigned to stepped care or stratified care groups, and who did not deliver the treatment that patients were allocated to after initial assessments. There is no precedent for this type of trial in this setting, so it is not known if clustering effects are relevant for *assessing clinicians*, nor do we have any prior information to calculate an intra-cluster correlation coefficient. Given the novel and pragmatic nature of this study, we have therefore followed conventional sample size calculation methods, and decided to control for clustering using multilevel-modelling *only if the random effect for the cluster level is statistically significant and improves model-fit*. This would enable us to examine if clustering effects are relevant or not in this context.

© 2021 Delgadillo J et al. *JAMA Psychiatry.*

Overall, we aimed to recruit a minimum of 10 therapists that carry out routine assessments in IAPT services. Between them, we expected that they would assess at least 760 during a 1-year study period, which would require each therapist to assess 2 cases per week on average.

eMethods 3. Preliminary Model-Building and Examination of Cluster Effects

Methods

We applied multilevel modelling (MLM) nesting patients (level 1) within *assessing clinicians* (level 2). Consistent with conventional model-building guidelines (Raudenbush, 1993), the analysis was performed in sequential steps, starting with single-level models and eventually developing multi-level and covariate-adjusted models. This process aimed to compare goodness-of-fit between models in order to eventually arrive at the most parsimonious and best-fitting model, adhering to the pre-registered plan to only retain a nesting structure (i.e., two-level model) if the cluster effect was statistically significant and improved model fit. Model fit was examined after each modelling step by inspecting the standard error of regression coefficients and the -2 log-likelihood ratio (a smaller -2LL statistic indicates better model fit). The intracluster correlation coefficient (ICC) was also calculated at each step, as an index of variability in treatment outcomes attributable to the clustering variable (*assessing clinicians*). We retained and interpreted the best-fitting and most parsimonious model achieved through this stepwise process.

Four logistic regression models were produced in this process, where the dependent variable was the primary outcome – reliable and clinically significant improvement (RCSI) in depression (PHQ-9) symptoms. [A] The first was a two-level model that simply examined the nested structure of the data, entering a random intercept for the *assessing clinician* (level 2). [B] The second two-level model additionally adjusted for baseline PHQ-9 severity. [C] The third two-level model was fully-adjusted, controlling for baseline PHQ-9 and entering the Group variable (stratified care vs. stepped care). [D] The final model was also fully-adjusted (including baseline PHQ-9 and Group), but it had a single-level structure, not adjusting for the assessing clinician.

© 2021 Delgadillo J et al. *JAMA Psychiatry.*

Results

Model A. Unconditional two-level model examining the nested structure of the data

Fixed effects							
**Variables**	**B**	**SE**	**t**	***p***	**CI-low**	**Ci-high**	
Intercept	-.080	.105	-.759	.448	-.287	.127	
Random effects							
**Nesting level**	**Variance**	**SE**	**Z**	***p***	**CI-low**	**Ci-high**	**ICC**
Random effect (clinicians)	.118	.072	1.636	.102	.036	.393	.106
B = regression coefficient; SE = standard error; CI = 95% confidence intervals; ICC = intracluster correlation coefficient							
-2 log likelihood (3408.069)							

Model B. Conditional two-level model, with fixed effects for baseline PHQ-9

Fixed effects							
**Variables**	**B**	**SE**	**t**	***p***	**CI-low**	**Ci-high**	
Intercept	.832	.304	2.740	.006	.236	1.427	
Baseline PHQ-9	-.054	.0166	-3.251	.001	-.086	-.021	
Random effects							
**Nesting level**	**Variance**	**SE**	**Z**	***p***	**CI-low**	**Ci-high**	**ICC**
Random effect (clinicians)	.146	.083	1.764	.078	.048	.442	.127
B = regression coefficient; SE = standard error; CI = 95% confidence intervals; ICC = intracluster correlation coefficient							
-2 log likelihood (3427.620)							

Model C. Conditional two-level model, with fixed effects for baseline PHQ-9 and Group

Fixed effects							
**Variables**	**B**	**SE**	**t**	***p***	**CI-low**	**Ci-high**	
Intercept	.699	.319	2.189	.029	.072	1.326	
Baseline PHQ-9	-.055	.017	-3.302	.001	-.087	-.022	
Group	.278	.219	1.269	.205	-.152	.709	
Random effects							
**Nesting level**	**Variance**	**SE**	**Z**	***p***	**CI-low**	**Ci-high**	**ICC**
Random effect (clinicians)	.131	.081	1.622	.105	.039	.440	.116
B = regression coefficient; SE = standard error; CI = 95% confidence intervals; ICC = intracluster correlation coefficient							
-2 log likelihood (3430.580)							

Model D. Single-level model, with fixed effects for baseline PHQ-9 and Group

Fixed effects							
**Variables**	**B**	**SE**	**Wald**	***p***	**Odds Ratio**	**CI-low**	**Ci-high**
Intercept	.620	.294	4.458	.035	1.859		
Baseline PHQ-9	-.049	.016	9.124	.003	.952	.922	.983
Group	.334	.149	5.045	.025	1.396	1.043	1.868
B = regression coefficient; SE = standard error; CI = 95% confidence intervals; ICC = intracluster correlation coefficient							
-2 log likelihood (1098.683)							

© 2021 Delgadillo J et al. *JAMA Psychiatry.*

Interpretation

Goodness-of-fit deteriorated at each step of the multilevel modelling process, indicated by an increasing magnitude of the -2LL statistic from model A through to model C, whereas the most parsimonious single-level model [D] had the best goodness-of-fit (i.e., smallest -2LL statistic). Furthermore, the random effect for the assessing clinicians was not statistically significant in any of the multi-level models [A-C]. This indicated that there is no significant evidence of cluster effects on clinical outcomes in this sample.

Therefore, following our pre-registered plan, we retained and interpreted the most parsimonious (least complex) and best-fitting single-level logistic regression model [D]. According to this model, cases randomised to the stratified care group were significantly more likely to attain reliable and clinically significant improvement of depression symptoms after therapy (Odds Ratio = 1.40, p = 0.025).

© 2021 Delgadillo J et al. *JAMA Psychiatry.*

eMethods 4. Economic Analysis

Methods

The economic analysis evaluated the incremental cost-effectiveness of stratified versus stepped care, from a health services perspective. The overall treatment costs for each patient were calculated based on the observed number of sessions at each step of care. This was multiplied by the relevant hourly treatment cost using the NHS reference cost database (Curtis & Burns, 2018), taking into consideration that there are different salary costs for clinicians offering low and high intensity interventions. The cost of training participating clinicians to use the StratCare app was also included in the calculation. As in the primary analysis, the outcome in the cost-effectiveness analysis was post-treatment RCSI status in the PHQ-9 measure. Using logistic regression models, we predicted the probability of RCSI for stratified and stepped care pathways. The models were estimated using 1,000 bootstrap samples (Briggs, Wonderling, & Mooney, 1997). The results were expressed as incremental treatment costs in stratified care compared to stepped care, relative to the percentage change in probability of RCSI. The joint distribution of the difference in cost (stratified care minus stepped care) and the probability of RCSI were plotted on a cost-effectiveness plane. Finally, a cost-effectiveness acceptability curve (CEAC) was used to present the probability of stratified care being cost-effective, compared to stepped care, for a range of willingness-to-pay values for one additional case of RCSI.

Results

Economic analyses indicated that the average cost per patient was £248.48 in stratified care and £146.45 in stepped care. After adjusting for age, gender and case complexity, the incremental cost of the stratified care pathway £104.5 (95% CI: £67.5, £141.6; $p < 0.05$) per patient. Stratified care was associated with a 7.9% (95% CI: 0.62%, 15.2%; $p < 0.05$) increase in the probability of remission (RCSI) of depression symptoms compared to stepped care. Figure 3 (displayed in the main article, and also in the next page) presents the CEAC, which shows that the probability of stratified care being cost-effective, compared to stepped care, is 50% when the willingness-to-pay (WTP) threshold per additional case of reliable improvement is £1,320 – this is known as the incremental cost-effectiveness ratio. The probability of stratified care being cost-effective increases to 80% and 90% for the WTP values of £2,100 and £3,050, respectively. Figure 4 presents the cost-effectiveness plane, showing that the joint distribution of the difference in cost and the difference in probability of reliable improvement lies mostly in the top-right quadrant, i.e. stratified care is more costly and more effective.

© 2021 Delgadillo J et al. *JAMA Psychiatry.*

eFigure 1. Cost-effectiveness Acceptability Curve, Showing the Probability of Stratified Care Being Cost-effective for a Range of Willingness-to-Pay Threshold for 1 Additional Case of Reliable Improvement*

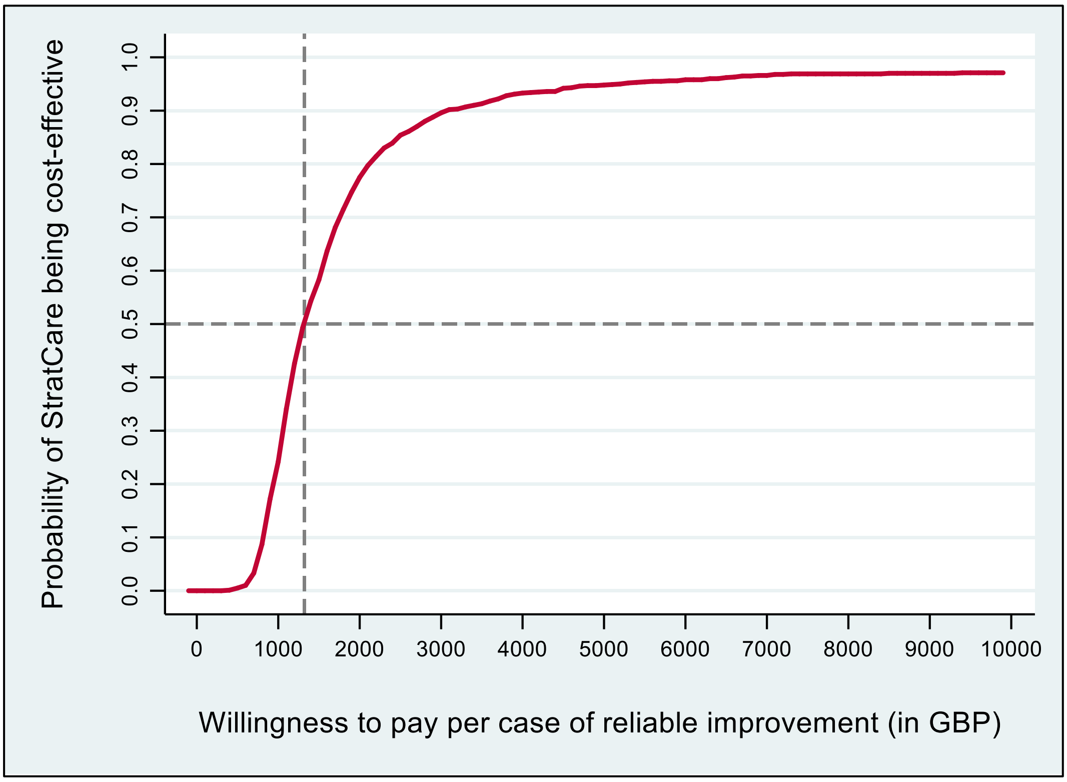

* Probability of Stratified Care being cost-effective (compared to Stepped Care model) is >50% if the WTP is >£1,320 per additional case of reliable improvement.

© 2021 Delgadillo J et al. *JAMA Psychiatry.*

eFigure 2. Cost-effectiveness Plane, Showing Incremental Difference in Cost and Probability of Reliable Improvement Between Stratified Care and Stepped Care Models§

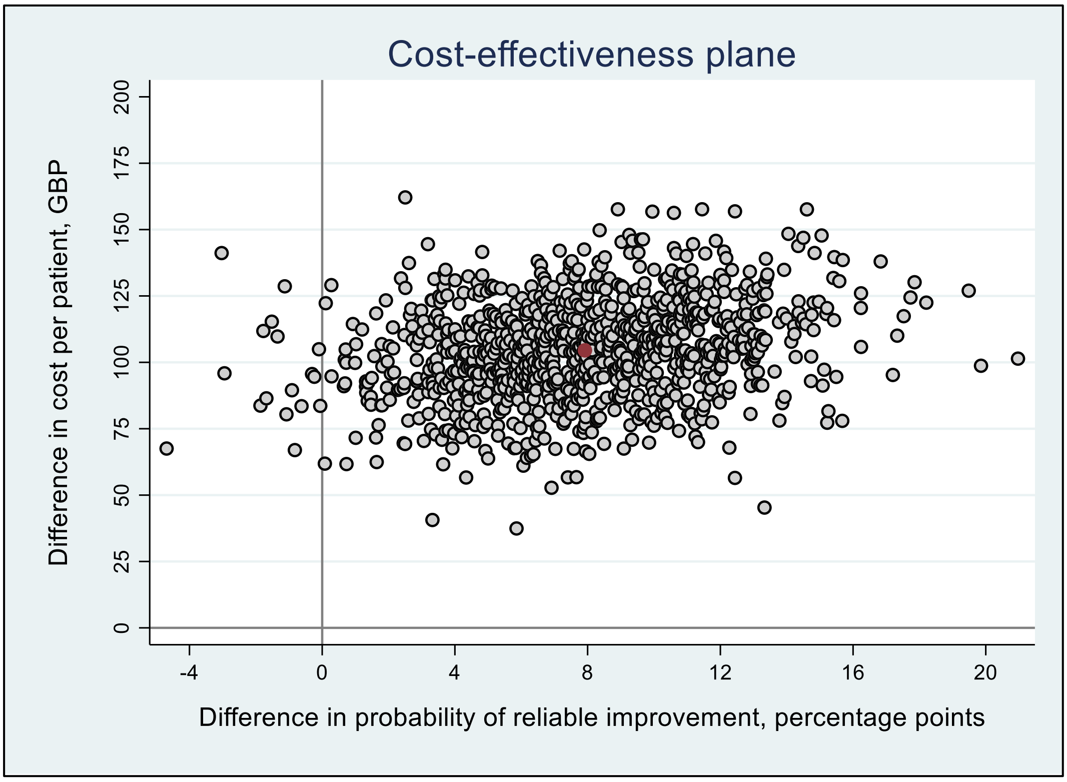

§ The red dot in the middle represents the mean difference in cost (£104.5) and mean difference in the probability of reliable improvement (7.9 percentage points)

© 2021 Delgadillo J et al. *JAMA Psychiatry.*

eReferences.

Bennett-Levy, J., Richards, D. A., Farrand, P., Christensen, H., Griffiths, K. M., Kavanagh, D. J., Klein, B., Lau, M.A., Proudfoot, J., Ritterband, L., White, J., & Williams, C. (Eds.). (2010). *Oxford guide to low intensity CBT interventions*. Oxford, UK: Oxford University Press

National Collaborating Centre for Mental Health. (2018). *The Improving Access to Psychological Therapies Manual*. https://www.england.nhs.uk/wp-content/uploads/2018/06/the-iapt-manual.pdf

National IAPT Team. (2015). *National curriculum for the education of Psychological Wellbeing Practitioners, Third edition*. London: NHS England/Department of Health.

Baldwin, S. A., & Imel, Z. E. (2013). Therapist effects: Findings and methods. In M. J. Lambert (Ed.), *Bergin and Garfield's Handbook of Psychotherapy and Behavior Change* (6th ed., pp. 258 – 297). New York: Wiley.

Delgadillo, J., Huey, D., Bennett, H., McMillan, D. (2017). Case Complexity as a Guide for Psychological Treatment Selection. *Journal of Consulting and Clinical Psychology, 85*, 835–853. https://psycnet.apa.org/doi/10.1037/ccp0000231

Fleiss, J. L., Levin, B., & Paik, M. C. (2013). *Statistical methods for rates and proportions*. John Wiley & Sons.

Raudenbush, S. W. (1993). Hierarchical linear models and experimental design. In L. Edwards (Ed.), *Applied analysis of variance in behavioral science* (pp. 459-496). New York: Marcel Dekker.

Briggs, A. H., Wonderling, D. E., & Mooney, C. Z. (1997). Pulling cost-effectiveness analysis up by its bootstraps: a non-parametric approach to confidence interval estimation. *Health Economics, 6*(4), 327-340. https://doi.org/10.1002/(SICI)1099-1050(199707)6:4%3C327::AID-HEC282%3E3.0.CO;2-W

Curtis, L. & Burns, A. (2018). *Unit Costs of Health and Social Care 2019*. Canterbury: Personal Social Services Research Unit, University of Kent. doi: 10.22024/UniKent/01.02.79286

© 2021 Delgadillo J et al. *JAMA Psychiatry.*
