# Supplementary material for: Stratified Care vs Stepped Care for Depression: A Cluster Randomized Clinical Trial
Source: JAMA Psychiatry. 2021 Dec 8;79(2):1–9. doi: 10.1001/jamapsychiatry.2021.3539 (PMC8655665; doi:10.1001/jamapsychiatry.2021.3539)
Supplement: Supplement 3. — Data Sharing Statement [file jamapsychiatry-e213539-s003.pdf]

Data Sharing Statement

Delgadillo. Stratified Care vs Stepped Care for Depression. *JAMA Psychiatry*. Published December 08, 2021. doi:10.1001/jamapsychiatry.2021.3539

Data
Data available: Yes
Data types: Deidentified participant data
How to access data: In line with the requirements of the research ethics committee that approved this research, requests for access to data are to be made in writing to the corresponding author. Only de-identified participant data in an encrypted file can be made available, along with a data dictionary, to suitably qualified researchers who (a) obtain ethical approval for their proposed analysis; (b) pre-register their statistical analysis plan; (c) provide a signed data-sharing contract which enables data storage and analysis for a time-limited period.
When available: With publication

Supporting Documents
Document types: None

Additional Information
Who can access the data: Suitably qualified academic researchers.
Types of analyses: Pre-registered statistical analyses.
Mechanisms of data availability: Suitably qualified academic researchers who (a) obtain ethical approval for their proposed analysis; (b) pre-register their statistical analysis plan; (c) provide a signed data-sharing contract which enables data storage and analysis for a time-limited period.
